# Determinants of Delayed Leprosy Diagnosis in an Endemic Area of Northeast Brazil: A Cross-Sectional Study

**DOI:** 10.1590/0037-8682-0230-2025

**Published:** 2025-12-15

**Authors:** William Lucas da Silva Mendes Pina, Lorena Ferreira de Azevedo Melo, Héllen Néo da Rocha, Juliana de Moura, Leonardo Lopes-Luz, Lívia Silveira Silva, Sálvia Cely Cerqueira Carvalho, Glicya Monaly Claudino dos Santos, Victor Santana Santos

**Affiliations:** 1 Universidade Federal de Sergipe, Departamento de Medicina, Lagarto, SE, Brasil. Universidade Federal de Sergipe Departamento de Medicina Lagarto SE Brasil; 2 Universidade Federal do Paraná, Curitiba, PR, Brasil. Universidade Federal do Paraná Curitiba PR Brasil; 3 Universidade Federal de Goiás, Instituto de Patologia Tropical e Saúde Pública, Goiânia, GO, Brasil. Universidade Federal de Goiás Instituto de Patologia Tropical e Saúde Pública Goiânia GO Brasil; 4 Universidade Federal de Goiás-Merck S/A Alliance, Innovation Hub in Point of Care Technologies, Goiânia, GO, Brazil. Universidade Federal de Goiás Merck S/A Alliance, Innovation Hub in Point of Care Technologies Goiânia GO Brazil; 5 Universidade Federal de Sergipe, Programa de Pós-Graduação em Ciências da Saúde, Aracaju, SE, Brasil. Universidade Federal de Sergipe Programa de Pós-Graduação em Ciências da Saúde Aracaju SE Brasil; 6 Universidade Federal de Sergipe, Programa de Pós-Graduação em Ciências Aplicadas à Saúde, Lagarto, SE, Brasil. Universidade Federal de Sergipe Programa de Pós-Graduação em Ciências Aplicadas à Saúde Lagarto SE Brasil

**Keywords:** Leprosy, Diagnosis, Delayed diagnosis, Brazil

## Abstract

**Background:**

Delayed diagnosis remains a primary challenge in leprosy control, often resulting in severe physical impairment and significant psychological distress among those affected. The factors contributing to this delay are complex and multifactorial, encompassing both patient-related and healthcare system-related elements. To investigate the patient- and health system-related factors contributing to delayed diagnosis of leprosy in an endemic area of Northeast Brazil.

**Methods:**

This cross-sectional study included 247 leprosy patients. Demographic and clinical characteristics, along with data on patient- and health system-related determinants associated with delays in diagnosis, were collected. Univariate and multivariate Poisson regression analyses were performed to examine associations between the outcome (delay in months) and the independent variables.

**Results:**

The median time from the first healthcare visit to leprosy diagnosis was 15.0 months (interquartile range: 6.0-36.0). Approximately 17% of participants had grade 2 disability (G2D) at diagnosis. In the multivariate Poisson regression analysis, female sex, residence in rural areas, delayed healthcare seeking after symptom onset, lack of initial suspicion of leprosy, multiple referrals requiring four or more consultations before diagnostic confirmation, and prior misdiagnosis with treatment for another condition were independently associated with prolonged diagnostic delay.

**Conclusion:**

This study identified significant delays in leprosy diagnosis in Sergipe, Brazil, which may contribute to the persistently high proportion of new cases with G2D. Both patient- and health system-related factors were associated with longer diagnostic delays. Urgent interventions are warranted to increase disease awareness among the general population and strengthen the capacity of primary healthcare services.

## INTRODUCTION

Leprosy is a chronic infectious disease caused by *Mycobacterium leprae* and *Mycobacterium lepromatosis*, primarily affecting the skin and peripheral nerves^1^^-^^4^. Despite advances in recent decades, leprosy remains a significant public health challenge in several countries, including Brazil^5^. One major obstacle to leprosy control is delayed diagnosis, which can lead to severe physical disabilities and psychological distress among affected individuals^1^^-^^3^^,^^6^^,^^7^.

The factors associated with delayed diagnosis are multifaceted, involving both patient- and healthcare system-related determinants. Barriers such as limited knowledge among healthcare professionals, resulting in procedural errors such as improper intradermal scraping collection, stigma and discrimination associated with the disease that may discourage patients from seeking care, and difficulties accessing healthcare services owing to the centralization of specialist professionals, particularly for complex procedures like biopsies under anesthesia, have been identified as key contributors to diagnostic delays^8^^-^^10^. However, there remains a lack of comprehensive studies that simultaneously evaluate both individual and systemic factors influencing the timeliness of leprosy diagnosis.

In Brazil, leprosy remains endemic across several regions, exhibiting heterogeneous spatial distribution^11^. Given this geographic variability, region-specific investigations are crucial to identify local determinants of diagnostic delay and generate evidence for targeted public health interventions. Our research group previously conducted a study in the state of Alagoas, identifying key factors associated with diagnostic delays in that setting^12^. Expanding this investigation to other endemic areas is crucial to understand broader patterns and inform disease control strategies.

In this study, we analyzed patient- and healthcare system-related factors contributing to delayed leprosy diagnosis in the state of Sergipe, a region in Northeast Brazil with persistent endemicity. 

## METHODS

### Study design and participants

This cross-sectional study included individuals aged ≥15 years diagnosed with leprosy between 2015 and 2022 in Aracaju, Sergipe, Northeast Brazil. Aracaju, the capital of Sergipe, has a population of approximately 630,000 inhabitants. The Leprosy and Tuberculosis Reference Center, located in Aracaju, provides specialized care for the entire Sergipe population, estimated at 2.2 million individuals. In 2021, the incidence rate of new leprosy cases in Aracaju was 11.45 per 100,000 inhabitants, and the proportion of grade 2 disability (G2D) among these cases was 11.9%, a figure indicating high endemicity according to Brazilian Ministry of Health criteria.

All consecutive patients who visited the Leprosy and Tuberculosis Reference Center in Aracaju between September 2022 and October 2024, either receiving multidrug therapy or returning for follow-up after previous treatment, were invited to participate. Participants with cognitive impairments that precluded comprehension of self-reported questions or with incomplete clinical data in their medical records were excluded. To minimize recall bias, individuals diagnosed before 2015 were also excluded.

### Sample size

A sample of 247 individuals affected by leprosy was required to determine the factors contributing to delayed diagnosis. The sample size was calculated based on the number of patients registered at the Leprosy and Tuberculosis Reference Center in Aracaju between 2020 and 2024 (N = 680), assuming that 50% of individuals would have a delayed diagnosis of leprosy, with a type I error (α) of 5% and a 95% confidence interval (CI 95%).

### Questionnaires and procedures

After providing written informed consent, participants were interviewed using a structured questionnaire adapted from previous studies^12^^,^^13^ to collect sociodemographic and clinical information, as well as to explore individual- and healthcare system-related factors that might contribute to diagnostic delays. Participants were asked about the onset of signs and symptoms, the time elapsed between symptom onset and seeking healthcare services, and the time between the first healthcare visit and definitive leprosy diagnosis.

Sociodemographic and clinical data included sex, age, years of education, place of residence (urban or rural), operational classification (paucibacillary [PB] or multibacillary [MB]), clinical form, disability grade, and presence of leprosy reactions. Participants were classified as having PB leprosy if they presented with ≤5 skin lesions, involvement of only one nerve trunk, or both, or if slit-skin smear microscopy was negative. MB leprosy was defined by >5 skin lesions, involvement of more than one nerve trunk, or both, or by positive smear microscopy findings^14^. Clinical forms were categorized as indeterminate (I), tuberculoid (TT), borderline (B), and lepromatous (LL)^15^. Disability was graded according to World Health Organization criteria: grade 0 indicated no disability; grade 1 represented loss or reduction of sensation in the eyes, hands, and/or feet without visible deformities; and grade 2 denoted loss of sensation accompanied by visible deformities^16^. Nerve involvement was defined as the presence of pain or nerve thickening on palpation, sensory loss detected by the monofilament test, or motor impairment^16^. Leprosy reactions were classified as type 1, characterized by acute inflammation of skin lesions or nerves, and type 2, defined by the appearance of inflamed cutaneous nodules with or without neuritis^17^. All clinical information was verified through a review of participants' medical records and/or notification forms in the *Sistema de Informação de Agravos de Notificação* (SINAN).

### Outcome

The outcome variable was the time (in months) elapsed from the first healthcare visit to definitive leprosy diagnosis.

### Data analysis

Categorical variables were described using frequencies and corresponding percentages. The assumptions of normality and homoscedasticity were assessed using the Shapiro-Wilk and Levene tests, respectively. Given the skewed distribution of most variables, nonparametric tests were used to assess differences between study groups. To examine factors associated with diagnostic delay, we conducted bivariate and multivariate Poisson regression analyses, modeling the outcome variable (delay in months) as a function of the independent variables. Disability grade was excluded from these models, as it is commonly used as a proxy for diagnostic delay. The significance level was set at 0.05. All analyses were performed using Stata version 14.0 (StataCorp LP).

### Ethical approval

The study was reviewed and approved by the Human Research Ethics Committee of the Universidade Federal de Sergipe (CAAE: 47713121.9.0000.5546). All procedures were conducted in accordance with the Declaration of Helsinki, and informed written consent was obtained from all participants. For minors, parents or guardians provided written informed consent, and minors provided their signature on the assent form.

## RESULTS

A total of 247 individuals affected by leprosy were included. The median age was 49 years (interquartile range [IQR]: 38.0-59.0). Most participants were male (149; 60.3%), had between 0 and 4 years of education (207; 83.8%), self-identified as mixed race (*Pardo*) (144; 58.4%), and resided in urban areas (180; 72.9%). Regarding disability grade at diagnosis, 86 (34.8%) had grade 0, 66 (26.7%) had grade 1, and 43 (17.4%) had grade 2. In terms of operational classification, 216 (87.5%) individuals were diagnosed with MB leprosy. The most frequent clinical form was LL, identified in 102 (41.3%) participants. Two hundred and sixteen individuals underwent smear microscopy, with 151 (61.1%) testing positive. A total of 179 (72.5%) participants experienced leprosy reactions, including 120 (67.0%) with type 1 (reversal reaction), 49 (27.4%) with type 2 (erythema nodosum leprosum), and 10 (5.6%) with mixed reactions (both type 1 and type 2) (Table 1).

**TABLE 1:** Demographic and clinical characteristics of people affected by leprosy assisted at the state reference center, Sergipe, Brazil, from 2022 to 2024.

Variable	n (%)
**Sex**	
Male	149 (60.3)
Female	98 (39.7)
**Age group (years)**	
15-19	7 (2.8)
20-29	20 (8.1)
30-39	43(17.4)
40-49	58 (23.5)
50-59	59 (23.9)
≥60	60 (24.3)
**Ethnicity**	
White	47 (19.0)
Black	51 (20.6)
Mixed (Pardo)	144 (58.4)
Indigenous	5 (2.0)
**Schooling (years)**	
0-4	207 (83.8)
5-8	40 (16.2)
**Residence zone**	
Urban	180 (72.9)
Rural	67 (27.1)
**Physical disability grade at diagnosis**	
Grade 0	86 (34.8)
Grade 1	66 (26.7)
Grade 2	43 (17.4)
Not reported	52 (21.1)
**Operational classification**	
Paucibacillary	22 (8.9)
Multibacillary	216 (87.5)
Not reported	9 (3.6)
**Clinic form**	
Indeterminate	12 (4.9)
Tuberculoid	16 (6.5)
Borderline	87 (35.2)
Lepromatous	102 (41.3)
Primary neural	8 (3.2)
Not reported	22 (8.9)
**Smear microscopy**	
Negative	65 (26.3)
Positive	151 (61.1)
Not performed	31 (12.6)
**Leprosy Reaction**	
Yes	179 (72.5)
No	68 (27.5)
**Type of Leprosy reaction (N = 179)**	
Type 1	120 (67.0)
Type 2	49 (27.4)
Type 1 and 2	10 (5.6)

*IQR: Interquartile range.

The median time (IQR) from the onset of signs and symptoms to seeking care at a healthcare facility was 6.0 months (1.0-12.0), ranging from a minimum of 1 month to a maximum of 24 months. The median time (IQR) from the first healthcare visit to confirmed leprosy diagnosis was 15.0 months (6.0-36.0), with a minimum of 0 months (same day) and a maximum of 250 months.

Table 2 presents the patient-related factors associated with diagnostic delay. A total of 128 (51.8%) participants reported not seeking medical care immediately after noticing their first signs and symptoms, with 107 (83.6%) believing their symptoms were not significant. Additionally, 44 (18.0%) participants suspected they had leprosy before visiting a healthcare facility.

**TABLE 2:** Potential delay factors related to people affected by leprosy assisted at the state reference center, Sergipe, Brazil, from 2022 to 2024.

Variables	n (%)
**Seeking healthcare facility immediately after notice of first symptom**	
Yes	119 (48.2)
No	128 (51.8)
**Reasons for not seeking care immediately after notice of first symptom**	
Did not think the symptoms are important enough to seek care	107 (83.6)
Did not notice the signs and symptoms	10 (7.8)
Live far from healthcare facility	3 (2.3)
Lack of money	2 (1.6)
Fear of being diagnosed by some serious illness	2 (1.6)
Fear of stigma	1 (0.8)
Others	3 (2.3)
**Participant suspected leprosy before diagnosis by health professional**	
Yes	44 (17.8)
No	203 (82.2)

Potential healthcare system-related factors contributing to diagnostic delay are outlined in Table 3. Most participants (238; 96.4%) were attended to at the first healthcare facility they visited. However, 79 (32.0%) were initially misdiagnosed and treated for other conditions, most commonly dermatological disorders (61.5%). A total of 157 (63.6%) participants reported being referred from primary healthcare (PHC) to a specialized service, with 143 (57.8%) referred to dermatologists. Regarding the number of consultations before a leprosy diagnosis was established, 41 participants (16.6%) had one consultation, 88 (35.6%) had two, 42 (17.0%) had three, and 76 (30.8%) required more than four consultations. PHC doctors most frequently suspected leprosy (111; 44.9%); however, diagnostic confirmation was primarily made by specialized physicians (142; 57.5%), particularly dermatologists.

**TABLE 3:** Potential factors related to the health service and professionals according to people affected by leprosy assisted at the state reference center, Sergipe, Brazil, from 2022 to 2024.

Variable	n (%)
**Participant was attended at the first healthcare service**	
Yes	238 (96.4)
No	9 (3.6)
**Reason for not having received health care (N = 9)**	
Lack of a physician	2 (22.2)
Others	7 (77.8)
**Initial misdiagnosis and mistreatment**	
Yes	79 (32.0)
No	168 (68.0)
**Misdiagnosed conditions**	
Dermatological conditions	48 (61.5)
Rheumatological conditions	11 (14.1)
Orthopaedic conditions	1 (1.3)
Other neglected tropical disease	8 (10.3)
Did not know	10 (12.8)
**Received a diagnosis of leprosy at the first healthcare visit**	
Yes	84 (34.0)
No	163 (65.9)
**No. of healthcare services attended before the diagnosis of leprosy**	
1	84 (34.0)
2	90 (36.4)
3	39 (15.8)
≥4	34 (13.8)
**Referred to a specialist**	
Yes	157 (63.6)
No	90 (36.4)
**Specialist who has been referred**	
Dermatologist	143 (57.8)
Neurologist	4 (1.7)
Orthopedist	4 (1.7)
Angiology / Vascular Surgeon	2 (0.8)
Ophthalmologist	2 (0.8)
Allergy specialist	1 (0.4)
Cardiologist	1 (0.4)
**No. of consultations necessary to receive the diagnosis of leprosy**	
1	41 (16.6)
2	88 (35.6)
3	42 (17.0)
≥4	76 (30.8)
**Health professional suspected leprosy**	
Medical doctor from Health Family Strategy	111 (44.9)
Dermatologist	94 (38.1)
Other medical specialities	24 (9.7)
Nurse from Health Family Strategy	18 (7.3)
**Health professional who confirmed the diagnosis of leprosy**	
Dermatologist	142 (57.5)
Medical doctor from Health Family Strategy	86 (34.8)
Other medical specialities	14 (5.7)
Nurse from Health Family Strategy	5 (2.0)

Supplementary Table 1 presents the median and IQR of the time elapsed between the first healthcare visit and definitive leprosy diagnosis among study participants. Overall, males exhibited a median delay of 14.0 days (IQR: 6.0-28.0), slightly shorter than that observed among females (16.5; IQR: 7.0-40.4). The diagnostic delay tended to be longer among individuals aged 30-39 years (19.0; IQR: 5.0-38.0) and those with lower education (0-4 years: 20.5; IQR: 7.0-39.0). Regarding ethnicity, the longest delay was observed among Indigenous participants (27.0; IQR: 10.0-114.0). Paucibacillary cases presented a higher median delay (26.0; IQR: 3.5-44.5) compared with multibacillary cases (14.0; IQR: 7.0-36.0). Participants who did not seek medical care immediately after noticing symptoms had a median delay of 24.0 days (IQR: 10.0-42.3). Similarly, those who experienced an initial misdiagnosis presented a longer time to diagnosis (20.0; IQR: 8.0-39.0) compared with participants without misdiagnosis (13.0; IQR: 6.0-32.0).

The multivariate Poisson regression analysis revealed that women had a 1.16-fold higher risk of delayed diagnosis compared with men (adjusted Incidence Rate Ratio [IRR] = 1.16; 95% CI: 1.10-1.22; p < 0.001). Individuals aged 40-49 years had a 1.34-fold higher risk of delayed diagnosis, while those aged 50-59 years had a 1.90-fold higher risk compared with individuals aged 15-19 years. Participants with lower educational attainment (0-4 years of schooling) had a significantly higher risk of delayed diagnosis (adjusted IRR = 1.39; 95% CI: 1.30-1.50; p < 0.001), as did those residing in rural areas (adjusted IRR = 1.26; 95% CI: 1.19-1.34; p < 0.001). Additionally, individuals with tuberculoid, borderline, or primary neural clinical forms exhibited an increased risk of diagnostic delay compared with those with the indeterminate form (Table 4).

**TABLE 4:** Multivariate Poisson regression showing factors associated with the time elapsed from first healthcare visit to definitive leprosy diagnosis among people assisted at the state reference center, Sergipe, Brazil, from 2022 to 2024.

	Bivariate analysis			Multivariate analysis (Pseudo R^2^ = 0.2375)		
Variable	IRR^a^	95% CI	P-value	IRR^a^	95% CI	P-value
**Sex**						
Female	1.10	1.03 to 1.14	<0.001	1.16	1.10 to 1.22	<0.001
Male	Reference	Reference	Reference	Reference	Reference	Reference
**Age group (years)**						
15-19	Reference	Reference	Reference	Reference	Reference	Reference
20-29	1.88	1.50 to 2.34	<0.001	0.92	0.73 to 1.16	0.495
30-39	1.96	1.59 to 2.42	<0.001	1.08	0.87 to 1.35	0.470
40-49	2.59	2.10 to 3.18	<0.001	1.34	1.08 to 1.67	0.007
50-59	2.85	2.31 to 3.50	<0.001	1.90	1.54 to 2.37	<0.001
≥60	1.53	1.24 to 1.89	<0.001	1.04	0.84 to 1.29	0.696
**Ethnicity**						
Black	Reference	Reference	Reference	Reference	Reference	Reference
White	1.48	1.38 to 1.59	<0.001	1.44	1.33 to 1.56	<0.001
Mixed (*Pardo*)	0.97	0.90 to 1.03	0.309	0.97	0.90 to 1.03	0.329
Indigenous	2.04	1.79 to 2.32	<0.001	1.06	0.92 to 1.24	0.413
**Schooling (years)**						
0-4	1.24	1.17 to 1.31	<0.001	1.39	1.30 to 1.50	<0.001
5-8	Reference	Reference	Reference	Reference	Reference	Reference
**Local of the residence**						
Rural	1.13	1.07 to 1.19	<0.001	1.26	1.19 to 1.34	<0.001
Urban	Reference	Reference	Reference	Reference	Reference	Reference
**WHO Operational classification**						
Paucibacillary	1.22	1.13 to 1.31	<0.001	0.95	0.86 to 1.05	0.342
Multibacillary	Reference	Reference	Reference	Reference	Reference	Reference
**Clinical form**						
Indeterminate	Reference	Reference	Reference	Reference	Reference	Reference
Tuberculoid	1.90	1.69 to 2.26	<0.001	1.77	2.26 to 3.45	<0.001
Borderline	1.52	1.34 to 1.73	<0.001	1.32	1.59 to 2.37	<0.001
Lepromatous	1.30	1.14 to 1.48	<0.001	1.15	0.99 to 1.31	0.056
Primary neural	2.08	1.78 to 2.44	<0.001	1.90	1.60 to 2.23	<0.001
**Seeking health care immediately after notice of first symptom**						
Yes	Reference	Reference	Reference	Reference	Reference	Reference
No	1.51	1.44 to 1.58	<0.001	1.68	1.59 to 1.77	<0.001
**Participant was attended in the first healthcare service**						
Yes	Reference	Reference	Reference	Reference	Reference	Reference
No	0.88	0.77 to 1.00	0.056	1.24	1.07 to 1.45	0.005
**Participant suspected leprosy before diagnosis by health professional**						
Yes	1.10	1.02 to 1.14	0.008	1.10	1.04 to 1.18	0.002
No	Reference	Reference	Reference	Reference	Reference	Reference
**Health professional suspected leprosy**						
Medical doctor from Health Family Strategy	Reference	Reference	Reference	Reference	Reference	Reference
Nurse from Health Family Strategy	1.31	1.17 to 1.45	<0.001	1.30	1.16 to 1.46	<0.001
Dermatologist	1.16	1.05 to 1.30	0.004	1.09	0.97 to 1.23	0.133
Other medical specialities	1.18	1.04 to 1.34	0.007	0.90	0.78 to 1.05	0.196
**No. of healthcare services attended before the diagnosis of leprosy**						
1	Reference	Reference	Reference	Reference	Reference	Reference
2	1.04	0.98 to 1.11	0.108	0.90	0.84 to 0.97	0.009
3	1.13	1.05 to 1.22	0.001	0.83	0.74 to 0.92	0.001
≥4	1.78	1.66 to 1.90	<0.001	1.39	1.26 to 1.52	<0.001
**No. of consultations necessary to receive the diagnosis of leprosy**						
1	Reference	Reference	Reference	Reference	Reference	Reference
2	1.24	1.15 to 1.35	<0.001	1.21	1.10 to 1.33	<0.001
3	1.01	0.92 to 1.11	0.751	0.86	0.77 to 0.96	0.010
≥4	2.13	1.98 to 2.31	<0.001	1.79	1.60 to 1.98	<0.001
**Initial misdiagnosis and mistreatment**						
Yes	1.37	1.30 to 1.43	<0.001	1.31	1.24 to 1.40	<0.001
No	Reference	Reference	Reference	Reference	Reference	Reference
**Health professional who confirmed the diagnosis of leprosy**						
Medical doctor from Health Family Strategy	Reference	Reference	Reference	Reference	Reference	Reference
Nurse from Health Family Strategy	1.52	1.30 to 1.77	<0.001	1.44	1.22 to 1.71	<0.001
Dermatologist	0.98	0.92 to 1.02	0.352	0.93	0.86 to 1.00	0.056
Other medical specialities	1.24	1.12 to 1.37	<0.001	1.73	0.88 to 1.34	<0.001

^a^ Incidence Rate Ratio (IRR). Pseudo R^2^ = 0.2289.

Participants who did not seek healthcare immediately after noticing their first signs and symptoms (adjusted IRR = 1.68; 95% CI: 1.59-1.77; p < 0.001), who were not attended to at the first healthcare facility visited (adjusted IRR = 1.24; 95% CI: 1.07-1.45; p = 0.005), who suspected leprosy before seeking care (adjusted IRR = 1.10; 95% CI: 1.04-1.18; p = 0.002), who required four or more referrals (adjusted IRR = 1.39; 95% CI: 1.26-1.52; p < 0.001), who needed four or more consultations for diagnostic confirmation (adjusted IRR = 1.79; 95% CI: 1.60-1.98; p < 0.001), and who were treated for another clinical condition (adjusted IRR = 1.31; 95% CI: 1.24-1.40; p < 0.001) were also more likely to experience diagnostic delay (Table 4).

## DISCUSSION

In this study, we observed a median time of six months from symptom onset to initial healthcare seeking and a median interval of 15 months from first healthcare contact to definitive leprosy diagnosis. Our findings highlight that key patient-related determinants of delayed diagnosis included female sex, rural residence, postponement of healthcare seeking after symptom recognition, and the patient’s own suspicion of leprosy. Factors within the healthcare system contributing to diagnostic delays encompassed lack of immediate evaluation at the initial point of contact, multiple referrals, the need for four or more consultations before diagnostic confirmation, and prior misdiagnosis with treatment for other clinical conditions.

Although previous studies have shown that men are more likely to experience delayed leprosy diagnosis owing to their tendency to neglect mild symptoms and reluctance to seek healthcare^18^^-^^20^, our study found that women were more likely to face diagnostic delays. This discrepancy may be explained by sociocultural factors influencing women’s healthcare-seeking behavior, such as limited access to services or socioeconomic barriers. Given that earlier research also highlights men’s reluctance to seek care^19^^,^^21^^,^^22^, healthcare professionals should remain alert to potential delays among both genders. It is essential that active case-finding and contact-tracing strategies be adapted to account for the distinct factors influencing healthcare-seeking behavior in men and women, ensuring timely diagnosis for all individuals.

Consistent with other studies, we found that individuals living in rural areas were more likely to experience delayed leprosy diagnosis. This finding aligns with the growing body of literature showing that geographic location significantly affects diagnostic timelines^9^^,^^22^^,^^23^. People in rural areas often face challenges such as limited healthcare access, scarcity of specialized professionals, and long travel distances to health facilities, all of which can delay diagnosis and treatment^24^. These barriers, combined with lower health literacy and the stigma surrounding leprosy, contribute to the prolonged time between symptom onset and diagnosis in rural populations^8^^,^^9^. Targeted public health interventions to improve healthcare access and awareness in these communities are therefore essential to reduce diagnostic delays.

Delayed healthcare seeking following the onset of early leprosy signs and symptoms was an important factor associated with diagnostic delay. Despite living in regions with high leprosy prevalence, many individuals fail to recognize early symptoms or delay seeking medical attention until the disease progresses^13^^,^^19^. Interestingly, individuals who suspected they had leprosy were paradoxically more likely to experience longer diagnostic delays, possibly owing to fear of stigma, which may lead them to conceal symptoms and avoid care^9^^,^^13^. Such behavior can worsen disease outcomes. Addressing this issue requires community education initiatives to increase awareness, combat stigma, and promote timely access to healthcare services.

A high number of additional consultations and referrals to specialized healthcare facilities were also significant factors contributing to diagnostic delay^8^^,^^9^^,^^13^^,^^25^. Although 34% of participants received their diagnosis at the first healthcare service they visited, in our study this variable referred to the first service attended, regardless of the number of consultations required. Thus, some patients were diagnosed at their first healthcare service but not during their first consultation, explaining the discrepancy between these variables and underscoring the complexity of the diagnostic pathway. This highlights the need for ongoing training in leprosy diagnosis and management, particularly in countries where the disease remains a public health concern, as well as investment in accurate diagnostic tools for use in primary healthcare units^1^. 

Since 2004, Brazil’s National Leprosy Control Program has aimed to decentralize care and strengthen PHC to improve early diagnosis and reduce disabilities^26^. However, reluctance toward decentralization persists in many Brazilian municipalities, and a high proportion of patients are still referred to specialized leprosy centers for diagnostic confirmation^27^. This continued reliance on specialized services may reflect the complexity and limitations of traditional diagnostic methods, such as smear microscopy and histopathology, which require invasive specimen collection, depend on highly trained professionals, and involve subjective interpretation of results. These challenges hinder their implementation in primary care settings^28^.

Additionally, one-third of our participants were misdiagnosed and received inappropriate treatment, indicating that healthcare professionals may fail to consider leprosy, even in endemic areas. This issue is compounded by the disease's variable clinical presentation, which can mimic other dermatological or neurological conditions^13^^,^^29^. Similar findings have been reported in studies from Colombia^13^, India^30^, and other regions of Brazil^12^^,^^19^. Although clinical evaluation remains central to leprosy diagnosis, especially in endemic settings, the high rate of diagnostic errors observed in our study reinforces the importance of supporting clinical suspicion with complementary diagnostic tools whenever possible. Anti-PGL-I serology and recent advances such as the rapid ML Flow test, incorporated into Brazil’s Unified Health System^31^, as well as highly specific molecular assays (quantitative polymerase chain reaction), offer valuable support for improving diagnostic accuracy^32^. While infrastructure and accessibility challenges persist, integrating these tools, rather than relying on a single method, can enhance early detection and reduce diagnostic errors, especially in cases with atypical or overlapping clinical presentations.

The high prevalence of the lepromatous form (41.3%) in this study contrasts with population-based data^33^ but aligns with findings from referral centers, where more severe cases are concentrated^34^. This overrepresentation likely reflects selection bias inherent to hospital-based samples, limiting generalizability and highlighting the importance of population-based studies to capture the full clinical spectrum of leprosy^34^.

This study has some limitations. It relies on participants' recollections, which introduces potential recall bias despite measures taken during data collection to minimize this effect. Our assessment of health system factors was based solely on patient-reported data, without input from healthcare professionals or institutional records. As a result, systemic shortcomings may not be fully captured, and provider perspectives are not represented. Additionally, this study was conducted among individuals affected by leprosy who received care at a reference center; therefore, the findings may not be directly generalizable to those managed exclusively in primary healthcare (PHC) settings. However, the results reflect the reality of many similar contexts in endemic areas, where reference centers remain the primary sites for leprosy diagnosis.

Although the final model demonstrated modest explanatory power (Pseudo R² = 0.2375), this outcome is expected given the complex, multifactorial nature of diagnostic delay. Even with a limited R², the model successfully identified key factors associated with delayed diagnosis and highlighted the need for further research to better understand and address these challenges. Moreover, there is currently no standardized threshold in the literature or from the WHO to define diagnostic delay in leprosy in terms of months. Consequently, our finding of a median delay of 15 months should be interpreted with caution and considered in relation to values reported in other settings rather than against a universally accepted benchmark. Future studies and international consensus efforts are needed to establish such definitions, ideally linked to the risk of disability and transmission associated with delayed diagnosis.

In conclusion, delays in leprosy diagnosis result from multiple factors involving both individuals and the healthcare system. Addressing these challenges requires coordinated action among healthcare providers, governmental agencies, and other stakeholders to enhance awareness and strengthen healthcare systems, ensuring timely diagnosis and treatment, particularly in leprosy-endemic regions such as Brazil. Strengthening primary healthcare should include capacity-building initiatives for early case detection, regular training of health professionals to recognize early signs of the disease, and integration of leprosy surveillance into routine PHC activities. Public awareness campaigns targeting both communities and health workers are also essential to reduce stigma and encourage timely healthcare seeking, thereby minimizing diagnostic delays and preventing disabilities.

## Data Availability

Data-available-upon-request: Research data is only available upon request.

## Appendix

**SUPPLEMENTARY MATERIAL TABLE 1:** Median and interquartile range of the time elapsed from first healthcare visit to definitive leprosy diagnosis assisted at the state reference center, Sergipe, Brazil, from 2022 to 2024.

Variable	Median (IQR)
**Sex**	
Female	16.5 (7.0-40.4)
Male	14.0 (6.0-28.0)
**Age group (years)**	
15-19	12.0 (2.0-22.0)
20-29	13.0 (6.0-32.0)
30-39	19.0 (5-38)
40-49	17.0 (7.0-42)
50-59	16.5 (9.0-48.0)
≥60	12.0 (2.0-25.0)
**Ethnicity**	
White	26.0 (10.0 to 41.0)
Black	13.0 (6.0 to 24.5)
Mixed (*Pardo*)	13.0 (6.0 to 34.0)
Indigenous	27.0 (10.0-114.0)
**Schooling (years)**	
0-4	20.5 (7.0-39.0)
5-8	13.0 (6.0-35.0)
**Local of the residence**	
Rural	17.0 (6.0-37.5)
Urban	14.5 (7.0-35.8)
**WHO Operational classification**	
Paucibacillary	26.0 (3.5-44.5)
Multibacillary	14.0 (7.0-36.0)
**Clinical form**	
Indeterminate	12.5 (7.75-32.5)
Tuberculoid	25.0 (9.75-46.5)
Borderline	14.5 (7.0-38.8)
Lepromatous	15 (6.0-34.8)
Neural pure	29.0 (14.0-50.0)
**Seeking health care immediately after notice of first symptom**	
No	24.0 (10.0-42.3)
Yes	10.0 (3.0-24.0)
**Participant was attended in the first healthcare service**	
No	22.0 (13.0-44.0)
Yes	15.0 (6.0-35.5)
**Participant suspected leprosy before diagnosis by health professional**	
No	15.5 (7.0-36.0)
Yes	17.0 (6.0-35.0)
**Health professional suspected leprosy**	
Nurse from Health Family Strategy	19.0 (11.5-38.5)
Medical doctor from Health Family Strategy	12.5 (4.25-37.3)
Dermatologist	15.5 (7.0-32.0)
Other medical specialities	13.0 (6.0-35.5)
**No. of healthcare services attended before the diagnosis of leprosy**	
1	-
2	18.0 (7.0-36.0)
3	22.0 (7.0-36.0)
≥4	20.0 (10.0-50.0)
**No. of consultations necessary to receive the diagnosis of leprosy**	
1	-
2	13.0 (6.0-35.0)
3	12.0 (3.0-29.0)
≥4	23.0 (10.0-48.0)
**Initial misdiagnosis and mistreatment**	
No	13.0 (6.0-32.0)
Yes	20.0 (8.0-39.0)
**Health professional who confirmed the diagnosis of leprosy**	
Nurse from Health Family Strategy	31.5 (19.0-56.8)
Medical doctor from Health Family Strategy	13.0 (5.0-36.5)
Dermatologist	15.0 (7.0-34.0)
Other medical specialities	25.0 (12.0-36.0)
